# Characterization of heat induced spherulites of lysozyme reveals new insight on amyloid initiation

**DOI:** 10.1038/srep22475

**Published:** 2016-03-01

**Authors:** Pankaj Sharma, Neha Verma, Pradip Kumar Singh, Suresh Korpole, Ashish

**Affiliations:** 1 CSIR-Institute of Microbial Technology, Chandigarh, India

## Abstract

Here, we report results obtained during our experiments to visualize how heat transforms globular protein, lysozyme into building block of β-amyloids. Light scattering experiments showed formation of lower order associated species around 50–70 °C followed by rapid cooperativity to β-amyloid fibrils. Interestingly, crystallization drops set at higher temperatures either led to aggregates or spherulites. The latter possess an amorphous β-fibril rich core with thin crystalline needles projecting outwards. Diffraction of the crystalline outgrowths revealed novel dimers and trimers of lysozyme where individual chains were similar to monomer with marginal gain in β-sheet content. Importantly, analysis of Amide I stretching frequencies showed that protein loses its secondary structure at temperatures higher than where we obtained crystals followed by rapid gain in β-sheet content. Interestingly, attempts to use the needles as seeds for more crystals led to “broom-like” fibril formations at the ends. Further, aggregation inhibitors like arginine and benzyl alcohol completely obliterated spherulites formation during crystallization. Refinement of crystals of lysozyme in presence of these molecules showed these small molecules bind to the interfaces of heat associated dimers and trimers. Overall our work concludes that heat induced weakly associated structures of lysozyme are the first step towards its amyloid formation.

It has remained intriguing how upon heating a globular protein like Hen Egg White Lysozyme rich in α/β-secondary structural content transforms into a building block of β-amyloids. Emerging biophysical data supports earlier notion that a portion of non-native and unstable conformations drive amyloid formation^1^,^2^,^3^. Prime limitation in precise characterization of the transition state is the polydisperse nature of the conformations and their association order which are in equilibrium prior to amyloid formation^4^. Out of the different protein candidates one can use for gaining atomic scale insight into the pre-amyloid state, we opted for lysozyme because its well-characterized monomeric globular form at low pH associates into β-amyloids upon heating^3^. Precedence teaches us that lysozyme adopts slightly disordered non-native like structures when subjected to higher temperatures^3^. Possibly, the disordered molecules nucleate to form protofibrils and higher order fibrillar structures^5^. Lara *et al.* showed that lysozyme undergoes hydrolysis at pH 2 and elevated temperatures close to 90 °C, and implied that the products of hydrolysis induce the amyloidic fibril formation^6^. Despite these reports, in absence of hydrolysis, it remains unclear how and when these non-native structures gain β-sheet architecture? Also, one wonders whether the non-native structures have to first associate to lose their native secondary structural content, and then gain β-sheet architecture characteristic of amyloid organization, or non-native structures first acquire β-sheet content at intrachain level which makes them competent to stack into filaments, or whether these events occur simultaneously. Since local concentrations of protein during amyloid formation are much higher than those used in biophysical experiments, it is not clear from previous reports how higher concentrations affect the structural changes in protein which accompany its transformation from globular monomeric state to be a unit in the amyloidic organization (Table S1).

Crystallization conditions offer high local protein concentrations, so we attempted crystallization of lysozyme under high temperature conditions to answer some of the above queries. In the crystallization drops, along with regular tetragonal crystals, spherulites were observed. Spherulites are sea-urchin like packing of molecules often obtained during attempts to crystallize protein. Spherulites are composed of microcrystalline as well as fibrillar patterns arranged in a radial fashion^7^. Spherulitic structures were also observed in case of bovine insulin under low pH (pH 2) and high temperature (65 °C) conditions as well as HEWL in the presence of sodium nitrate and sodium thiocyanate solutions under *in vitro* conditions^7^,^8^,^9^,^10^,^11^,^12^. Though amyloidic nature of bovine insulin spherulites has already been reported, but no report is available on extraction and structural analysis of the protein in the crystalline outgrowths of spherulites, neither for insulin nor for any other protein including lysozyme^7^,^12^,^13^. Formation of spherulites has been well studied in case of synthetic polymers like polyethene and crystallization of metals^14^,^15^, in biological systems even after five decades of initial reports of spherulites observed during protein crystallization, their detailed characterization remains unexplored^16^. Interestingly, spherulites with amyloidic origin/constitution have been reported in the mammary tumors of dogs and rat model of Alzheimer’s disease^17^,^18^. Connecting them with onset or progress of protein association based diseases, spherulites have also been observed in brain sections of patients suffering from a particular strain of Creutzfeldt-Jacob disease (CJD), in amyloid plaques of Down’s syndrome and Gerstmann-Sträusler-Scheinker diseases^19^,^20^. In this work, along with biophysical characterization of the heat induced association of lysozyme, we attempted to diffract the thin needles emerging out of the spherulite core. Results showed that upon increasing temperature, lysozyme molecules associate and loss of secondary structure as proposed earlier appears to occur after this initial step. Additionally, we show here that small molecules capable of interfering with the early association can obliterate spherulite formation.

## Results and Discussion

### Tracking the intermediate state in solution

**Figure 1:**
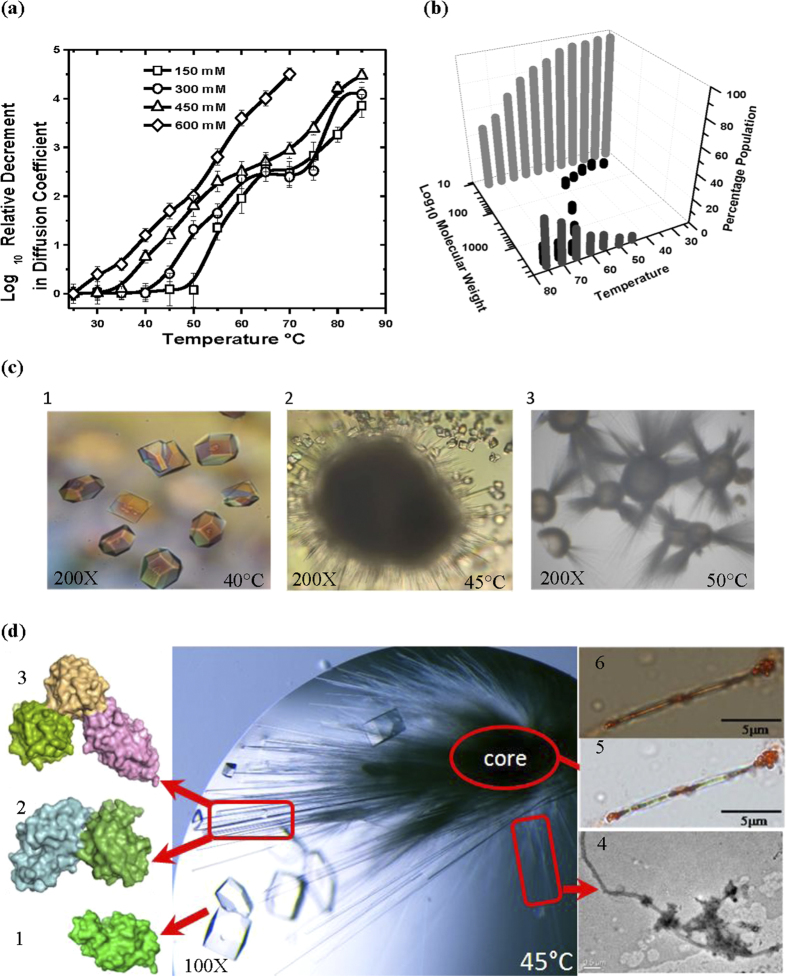
Tracking the increase in particle size of lysozyme as a function of heating. **(a)** Relative change in diffusion coefficient at 20 mg/ml protein concentration on heating lysozyme from 20 to 85 °C in 150 mM (-◻-), 300 mM (-○-), 450 mM (-Δ-) and 600 mM (-§-) NaCl at pH 3.8 is presented here. (*Lines are spline curves plotted to guide the eye on the trend*). **(b)** SEC-MALLS data as a function of temperature showing monomeric (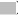), dimeric and trimeric associated states (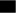), and higher order aggregates (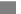) in solution of lysozyme incubated at different temperatures. **(c)** Crystallization of lysozyme at elevated temperatures. Tetragonal crystals at 40 °C **(1),** drop contained needle like crystals emerging out spherulite and normal tetragonal crystals at 45 °C **(2)**, spherulites formed in crystal drop at 50 °C **(3)**. **(d)** Crystallization drop containing spherulite and usual tetragonal crystals of HEWL formed at 45 °C. Tetragonal crystals were composed of monomeric protein **(1)**, needle shaped crystalline outgrowths were composed of natively associated protein molecules **(2,3)**, thin spikes analyzed using TEM were composed of amyloid fibrils **(4)**, samples taken from amorphous core get stained with Congo red dye **(5)** emit apple-green birefringence pattern when visualized under cross polarizers thus confirms that core is composed of amyloid fibrils **(6)**.

Previous reports have shown that the kinetics of lysozyme amyloid formation depends on temperature, protein and salt concentration^5^,^21^,^22^. Taking precedence from these studies, we monitored the effect of these parameters on relative change in the diffusion coefficient of lysozyme using dynamic light scattering (DLS). Diffusion coefficient was evaluated at three different protein concentrations (10, 20 and 30 mg/ml) with varying NaCl concentrations (150, 300, 450 and 600 mM) as a function of temperature ranging from 25 to 85 °C (Figs 1a and S1). Interestingly, patterns observed for lysozyme at 300–450 mM NaCl suggested that the transition from soluble to associated state follows a three-state process (Figs 1a and S1). Of the three states, intermediate state was found to be directly dependent on protein concentration and decrement in diffusion coefficient could be due to the formation of lower order associations within the temperature range of 40-(60–70 °C). Further rise in temperature lead to the formation of higher order aggregates. Previously, DLS and atomic force microscopy (AFM) based studies have shown that during HEWL aggregation under low pH (pH 2) and high temperature conditions (50 °C), monomers fuse to form oligomers which act as basic nucleating and growth unit to form the protofilaments^5^. Our results also revealed that at lower NaCl concentration (150 and 300 mM) onset of third state was delayed whereas at higher NaCl concentrations (600 mM) a clear observation of this intermediate step was obliterated (Figs 1a and S1). On the basis of decrement in diffusion coefficient values, it can be concluded that the average particle size increased as a function of temperature at all protein concentrations with increase in salt concentrations. Since DLS provides an average read-out for different species, unassociated or associated, we analyzed degree and state of association using gel filtration assembly connected to MALLS. Based on DLS experiments, a protein concentration of 20 mg/ml in buffer containing 450 mM of NaCl was used for carrying out this experiment. Samples pre-incubated at different temperatures were injected in gel filtration column and their retention time based mass values were estimated (Fig. 1b). Interestingly, we observed that protein mainly eluted as monomer till 35 °C, but a small population corresponding to dimer (~28 KDa) was also observed for sample incubated at 40 °C. The sample incubated at 45 °C showed populations corresponding to dimeric and trimeric state based on estimated mass. Alongside higher order associations also start forming at 45 °C and above. At higher temperatures (up to 55 °C), population of dimeric and trimeric associations increased. At 60 °C and above pronounced peaks starts appearing close to the void volume suggesting formation of higher order “irresolvable” state. Overall, SEC-MALLS experiments brought forth that upon heating portion of population of lysozyme molecules in solution gets associated to form dimer and trimer before forming higher order associations. Taking lead from DLS and SEC-MALLS we tried to crystallize lysozyme at temperatures 35 to 55 °C.

### Crystal Structures of Temperature Induced Lysozyme Dimer and Trimer

While on one hand, lysozyme forms heat (and alcohol) induced amyloids^23^,^24^, its monomeric form is well characterized. Before our work, crystals of lysozyme were grown up to 55˚C, but refined structure deposited in RCSB-PDB (PDB ID: 1BGI) is of the crystal grown at 37 °C and it is monomer^25^,^26^. We attempted crystallization of this protein at elevated temperatures (35–55 °C) with 0.9 to 1.6 M NaCl in mother liquid. At 40 °C, between 0.9–1.3 M NaCl mostly tetragonal crystals with standard unit cell parameters of HEWL (*i.e.* α = β = γ = 90° and a ≈ 79 Å, b ≈ 79 Å and c ≈ 37 Å). Small spherulite-like structures were formed in drops containing 1.2–1.3 M NaCl (Fig. 1c). Spherulites are star-shaped organization where thin projections emerge out of centre and a clear Maltase-cross extinction pattern could be observed between crossed polarizers^9^ (Fig. S2). Beyond 1.3 M NaCl, lysozyme aggregated at 40 °C and formed non-diffractable deformed crystals (Table S2). Interestingly, at 45 °C lysozyme crystallized into tetragonal and orthorhombic crystals and size of spherulites increased as compared to those seen at 40 °C. At 45 °C, in wells containing 1.2–1.3 M NaCl in mother liquid, only 1–2 spherulites were formed along with normal tetragonal and orthorhombic crystals in the corresponding crystallization drops (Fig. 1c). In these spherulites, needle shaped crystals varying in diameter (ranging from 35 to 15 μm) along with very thin non-crystalline “spikes” were observed emerging out of the core. These needle shape crystals were suitable for mounting for diffraction data collection. As per literature, this is the first work where crystalline growths were carefully abstracted from spherulites and examined for their diffraction quality (Fig. 1c). At NaCl concentration higher than 1.3 M at 45 °C, numerous spherulites of smaller size with very thin needle-like crystals were formed in the crystallization drops. A similar trend was observed in case of temperature induced spherulites of bovine insulin, where spherulite size decrease but their number increased with increase in NaCl concentration^8^. Repeatedly, we found that at 45 °C, lysozyme existed in three different states namely tetragonal/orthorhombic crystals, spherulite core and needle shaped crystals, suggesting that some kind of equilibrium exists between packing states under given experimental conditions. At temperatures higher than 45 °C, there was a pronounced formation of the spherulites or non-diffractable aggregation (Table S2).

**Figure 2:**
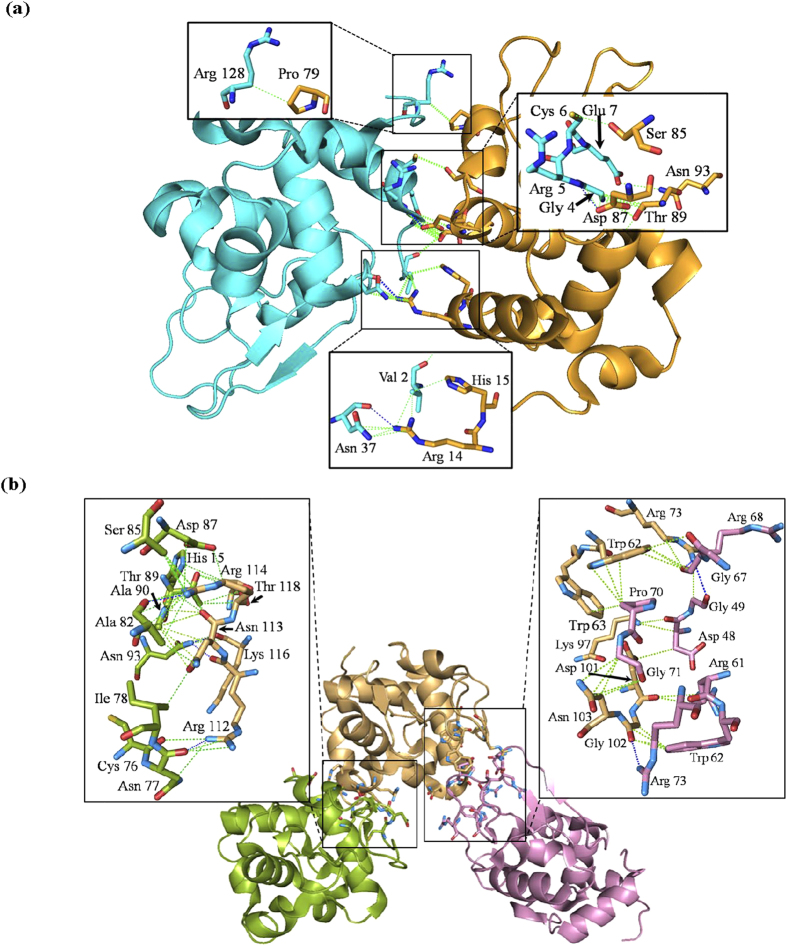
Structure of loosely associated dimeric **(a)** and trimeric state **(b)** of lysozyme at 45 °C. (*Insets*) Residues involved in (represented as sticks) interactions between protein chains. Dashed lines in blue represent hydrogen bonds while in green represent hydrophobic interactions, respectively.

Initial attempts to acquire diffraction data from needle-shaped crystals at room temperature by capillary mounting failed, either because crystals dehydrated during mounting or incident X-rays were damaged them. Analysis of partial images acquired under these conditions confirmed that the unit cell parameters in the needle-shaped crystals were different from all previously reported values (a = 31.6, b = 66.4 and c = 104.4 Å). Attempts were repeated to acquire diffraction data by using cryoprotectant (20% glycerol in mother liquid) at ~100 K. Importantly, after cryoprotection the unit cell parameters of crystals were identical to crystals diffracted at room temperature, thus cryoprotectant did not change/affect the cell parameters. Analysis of diffraction data from these crystals revealed that there were two and three lysozyme molecules in the asymmetric units (Fig. 1d). All the needle-like crystals broken from spherulites belonged to orthorhombic space group. The refinement of structures was initiated by molecular replacement (MR) method using PDB submission 1BGI as a search model^25^. Same protocol was employed to resolve structures of usual tetragonal and orthorhombic crystals (formed along with spherulites) which showed a single chain present in the crystal lattice. This structure was refined to R-factor and R-free factor of 18.22% and 22.98%, respectively at resolution of 1.71 Å (Table 1). Diameter of the needle shaped crystal abstracted from spherulite was 30 μm, which upon analysis showed two chains of protein in the asymmetric unit. This crystal belong to orthorhombic space group *P*2_1_2_1_2 having unit cell dimensions: a = 31.6, b = 66.4 and c = 104.4 Å. At 1.95 Å resolution, the data could be refined to an R-factor and R-free factor of 18.13% and 22.52%, respectively (Table 1). PDBSUM and PROT-PROT servers based analysis of the refined structure showed that the two chains in the asymmetric unit were weakly associated *via* two H-bonds and 22 non-bonded contacts not reported earlier in any lysozyme structure having two chains in the asymmetric unit (Tables S3–S5) (Fig. 2a)^27^. Particularly, Arg14 and Asp87 of Chain A forms H-bonds with Asn37 and Arg5 of Chain B, respectively (Table S3). The needle shaped crystal with three molecules in the asymmetric unit had a diameter of 25 μm. The resolution of this structure was 2.65 Å, crystal belongs to orthorhombic space group *P*2_1_2_1_2_1_ with unit cell dimensions: a = 31.4, b = 92.3 and c = 114.2 Å. This crystal was refined to an R-factor and R-free factor of 20.60% and 30.45%, respectively (Table 1). High R-free and R-merge values may be due to anisotropic diffraction or radiation damage due to X-rays. Refined structure of the trimeric state was also analyzed by PDBSUM and PROT-PROT servers for interchain interactions^27^. Results brought forth unique interactions between the protein chains which were not reported earlier (Tables S3 and S4). Total 47 interactions including four H-bonds and 43 non-bonded contacts were present between ten residues of Chain A and five residues of Chain B at the interface. Chain A residues: Asn77, Asn93 and Ala82 were involved in H-bonding with Arg112, Lys116 and Arg114 of chain B, respectively. Chain B was also weakly associated with Chain C through interaction between seven residues of Chain B and nine residues of Chain C. Analysis provided that there are three H-bonds between Arg73 and Gly102 residues of Chain B and Gly49, Arg68 and Arg73 of Chain C, respectively, besides 28 non-bonded interactions between these two chains (Tables S3 and S4) (Fig. 2B).

Analysis of the secondary structural content of lysozyme in monomeric, dimeric and trimeric states showed that α-helical and β-sheet content in monomeric and dimeric states were comparable to earlier known structures of native HEWL with ~41% helical and ~6% β-sheet content. However, PDBSUM and PROT-PROT based analysis computed only a fractional gain in the β-sheet content (1.8%) and decrease in helical content (0.7%), in the trimeric state due to the formation of two parallel β-strands made by residues Cys64-Asn65 and Ile78-Pro79 in addition to three anti-parallel β-strands (between residues 43–45, 51–53 and 58–59) known to date^27^. Moreover, it has also been suggested that high temperature and low pH induced unfolding of lysozyme starts at β-domain (residues 39–85) and the key residues involved in initiation of this process are Ile55, Ile58 and Asp66^2^,^3^. Interestingly, residues forming additional β-strands in our structure (Cys64-Asn65 and Ile78-Pro79) may lead to destabilization of β-domain due to their close proximity to the residues known to initiate unfolding process (Fig. S3). Further, we also observed that in the trimeric arrangement, chain C interacts with most of the residues which form the active site pocket of chain B. This interaction implies that if such an intermediate occurs in solution, it should reduce the bactericidal activity of this protein.

It is relevant to mention here that we also attempted to stain tetragonal crystals and three types of association states in spherulites *i.e.* the central core, thin spikes and needle shaped crystals with amyloid staining dyes, Thioflavin T (ThT) and Congo red (Figs 1d and S4). Only samples taken from spherulitic core have significantly enhanced fluorescence value compared to crystalline outgrowths and displayed typical apple green birefringence pattern under cross polarizers confirming that the core of spherulites were composed of amyloid fibers (Fig. 1d). Additionally, increase in fluorescence value at 440 nm was observed as a function of temperature upon adding ThT to a solution of lysozyme (Fig. S4). To understand nature of ‘thin non-crystalline spikes’ emerging out of spherulite core, we analyzed them on transmission electron microscopy (Fig. 1d). This technique has been extensively used by researchers to investigate HEWL amyloid fibrils^28^,^29^,^30^,^31^,^32^. The thin spikes had typical amyloid morphology as they were composed of unbranched fibrils having length more than  μm and a diameter of ~20–30 nm. Closer inspection shows that the fibrils were composed of protofilaments, wrapped around each other (Fig. S5). Since crystalline outgrowths (having diameter of 30–25 μm) were composed of weakly associated lower order oligomers and non-crystalline spikes were amyloidic in nature, these results support that as diameter of spherulitic outgrowths decreases more protein molecules associate together leading to the formation of protofilaments which may wrap around each other to form fibrils (present in thinner spikes and in core). Also, simultaneous formation of needle shaped crystals as well as amyloidic spikes from spherulites demonstrate that they may represent different stages of amyloid formation pathway.

### Intermediates Lack Secondary Structural Content and Bactericidal Activity

**Figure 3:**
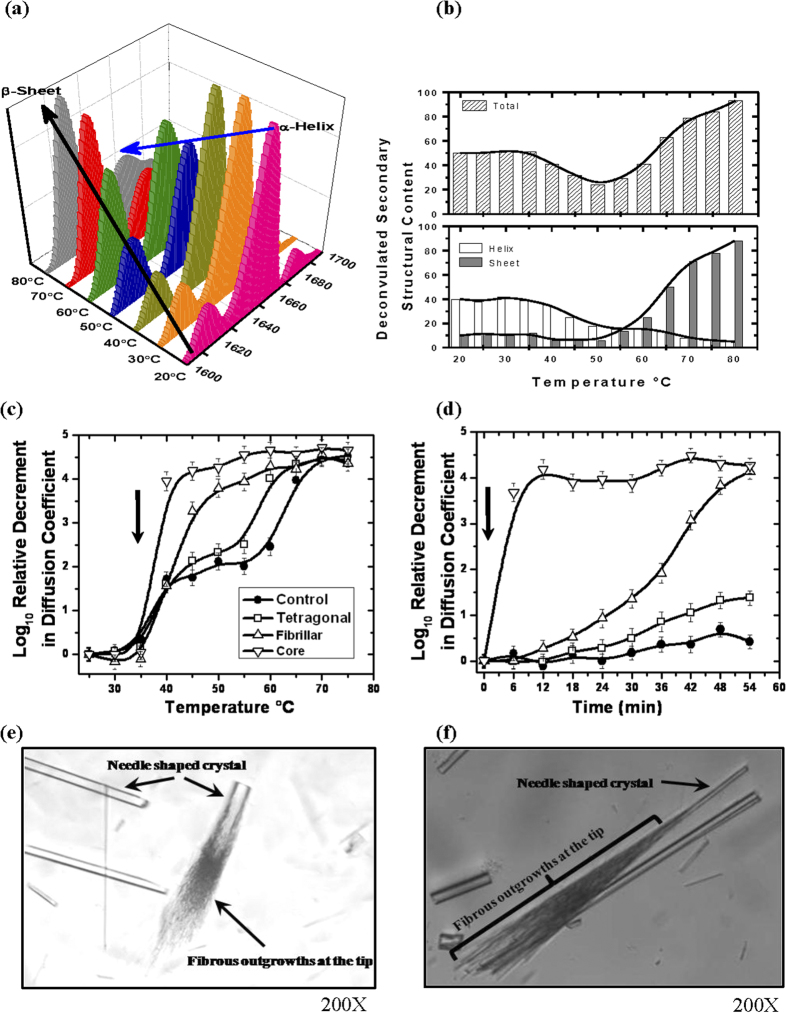
Effect of temperature on secondary structure and seeding on lysozyme **(a)** FTIR spectra of lysozyme (20 mg/ml) in buffer with 700 mM NaCl in the region of amid-1 band. Spectra shows changes in α-helical and β-sheet content as a function of temperature. **(b)** Deconvulation of the acquired FTIR data showed changes in total secondary content (*upper panel*) and, α-helix (◻s) and β-sheet (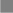) between 20 to 85 °C (*lower panel*). **(c)** DLS data of 20 mg/ml lysozyme without any seeds (-●-), seeded with crushed tetragonal crystals (-◻-), seeded with fibrillar crystals (-∆-) and seeded with white core (-∇-) as a function of temperature. Arrow represents the temperature at which seeds were incubated into solution. (*Lines are spline curves plotted to guide the eye on the trend*). **(d)** Tracking of relative decrement in the diffusion coefficient of lysozyme without any seeds (-●-), with seeds of: crushed tetragonal (-◻-), fibrillar crystals (-∆-) and seeds from core (-∇-) as a function of time. Arrow represents time of addition of crushed crystals or core. (*Lines are spline curves plotted to guide the eye on the trend*). (**e,f**) Two close-up images of the needle shaped crystals abstracted from spherulites to grow them further in lysozyme solution at 45 °C. “Broomy” or fibrillar outgrowths were seen on one end of these crystals support that these crystals are competent nucleation sites for fibrillar organization.

To determine the change in secondary structural content as a function of temperature, we opted for attenuated total reflection (ATR) FT-IR as this technique is not limited by protein and salt concentration. Protein and buffer conditions similar to those where spherulites were observed during crystallization attempts were used. Thus, spectra were collected for 2 mg/ml protein dissolved in buffer containing 700 mM NaCl, between 20–80 °C with 5 °C ramping. Analysis was focused on the Amide I region (1700–1600 cm^−1^) of the spectra as it is reflective of the secondary structure^33^,^34^. Deconvulation of the Amide I region for samples at different temperatures was done with emphasis on α-helical (~1655 cm^−1^) and β-sheet (~1620 and 1680 cm^−1^) contents (Figs 3a,b and S6). Summation of α-helical and β-sheet at each temperature during study suggested that combined content of these hydrogen-bonded secondary structural elements decreased in the temperature range of 45–60 °C. Interestingly, this zone corresponds to the temperature range where intermediate stage occurred in the DLS and SEC-MALLS experiments. Together these results suggest that protein molecules with native and non-native structures may be present in equilibrium within this temperature range. Such presence of native and non-native states was well supported by crystallization experiments where weakly associated species (with native structure) as well as non-native amyloid fibrils were observed simultaneously. Further increment in temperature during FTIR experiments led to decline in α-helical content and most of the secondary structural content was composed of β-sheets.

Since lysozyme is known for its bactericidal activity, which in turn is a reflection of retention of the functional shape of the protein, we performed cell survivability estimation with preheated samples (20 mg/ml) of lysozyme at increased temperatures (Fig. S7). Increased cell survivability supported loss in functional potency of the lysozyme molecule above 40 °C Previously, it had been reported that T_m_ of HEWL is 78 ± 0.5°C at pH 3.0, thus ruling out the possibility of compromised activity due to misfolding of lysozyme below its T_m_^23^. Reduced activity of protein at the median temperature range (between 40–65 °C) in conjunction with our results from DLS, SEC-MALLS, crystallization and FT-IR, is by virtue of its association which somehow affects availability of its active site also observed in the structure of weakly associated trimer.

### The Associated Crystals Are “On-pathway” Precursors

Based on above findings, one important query remains, whether the oligomer states of lysozyme are glimpses of events which occur prior to mature β-amyloid fibril formation or not ? To probe this, we incubated different types of crystals (washed with buffer prior to dissolving in the solution) and the white amyloid core (as seeds) in lysozyme solution (20 mg/ml), and tracked decrement in relative diffusion coefficient values of particles in solution (as done earlier). Comparable masses of crushed crystals (tetragonal and needle-shaped) and central white core were dissolved into solution of lysozyme at 35 °C. Repeated washing of crystals and needles ensured no portion of fibrillar core was present on their surface and thus experiments were unaffected by this possible contamination of higher order associations (Fig. 3c). DLS results clearly showed that in comparison to the control sample, where nothing was added, addition of tetragonal crystals showed a slight decrease in the particle size of protein probably due to higher amounts of protein in solution as observed during earlier DLS experiments. Interestingly, the trend in decrement in diffusion coefficient implied that addition of needle-shaped crystals or “core” act as seeds and converted the observed three-state process into a two-state process, possibly by “skipping” the need for the formation of intermediate state during temperature induced formation of lysozyme amyloids. To further substantiate our results, we also carried out this experiment as a function of time to see if these seeds can alter the kinetics of amyloid formation at a constant temperature. In this case, the “seeding” of lysozyme solution was done at 35 °C with different crystal forms and core, but the temperature was then maintained at 40 °C (Fig. 3d). While, the diffusion coefficient of molecules in control sample reduced slightly, addition of tetragonal crystal increased it a bit more, possibly due to increase in total protein concentration due to dissolution of crystals in solution. Importantly, addition of the needle-shaped crystals caused the decrement to occur rapidly. This affect was substantially pronounced in the samples where we added portions of the central white core. Ability of amyloid core to trigger a high order association was somewhat expected, but the trend shown by the needle-shaped crystals abstracted from spherulites supports that the temperature induced low order association *viz*. dimeric and trimeric state of lysozyme seen in the crystalline form represent on-pathway precursors of lysozyme amyloid formation.

### Attempts to re-seed thin needle shaped crystals

To improve size of the thin needle shaped crystals emerging from spherulites, we used them as seeds in droplets having 20 mg/ml of lysozyme. Essentially, broken needle shaped crystals were added to the drops of protein solution for crystallization at 35, 45 and 50 °C. At 35 °C, only tetragonal crystals were formed in the crystallization drops without any detectable change in the morphology of added needle shaped crystals. Interestingly at 45 °C, “broom-like” fibrous outgrowths at the tips of these crystals appeared after 8–10 days of incubation (Fig. 3e,f). A close inspection revealed that tips of thin needle shaped crystals act as seed for the formation of these fibrous outgrowths. Such branching has been shown earlier, but with no reference for amyloid formation^7^. Unfortunately, these fibrous outgrowths at ends of crystals were too delicate to separate and stain with Congo red. Use of these outgrowths as seeds for fresh crystallization at 45 °C led to the formation of spherulites (Congo red positive) without any needle shaped crystals within 2–3 days of crystallization. This further supports that thin needle shaped crystals are composed of “on-pathway” precursors of amyloid formation. At 50 °C, the thin needle seeds dissolved rapidly into the crystallization drops and spherulites were formed after approximately within 1–2 days.

### Arginine or Benzyl Alcohol abolish formation of spherulites

**Figure 4:**
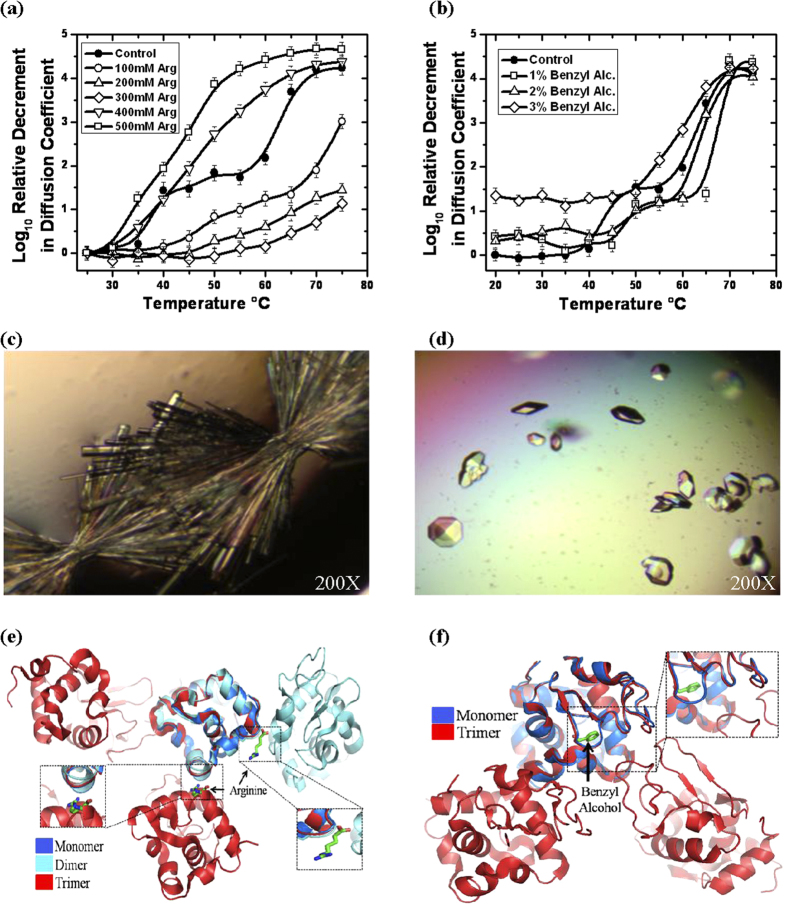
Effect of aggregation inhibitors on thermal association of lysozyme in solution. (**a**) Relative decrement in the diffusion coefficient of lysozyme without any arginine (-●-) and with: 100 mM (-○-), 200 mM (-△-), 300 mM (-◊-), 400 mM (-∇-) and 500 mM (-◻-) of arginine is plotted as a function of temperature. (*Lines are spline curves plotted to guide the eye on the trend*). (**b**) Temperature induced changes in the diffusion coefficient of lysozyme without benzyl alcohol (-●-), and with 1%(-◻-), 2% (-∆-) and 3% (-◊-) of benzyl alcohol is presented. (*Lines are spline curves plotted to guide the eye on the trend*). **(c)** Images of orthorhombic and tetragonal crystals of lysozyme formed in the presence of arginine and **(d)** benzyl alcohol at 45 °C. Importantly, spherulites were observed neither with arginine nor with benzyl alcohol crystal set-ups. **(e)** Structure of lysozyme with bound arginine and (F) benzyl alcohol superimposed with the heat induced dimeric and trimeric association of lysozyme. (*Insets*) Ligands are shown in stick representations.

Earlier, it has been reported that arginine and benzyl alcohol inhibit temperature induced amyloidosis of lysozyme^35^,^36^,^37^,^38^. Recently, it was demonstrated that 100–500 mM arginine significantly delays the aggregation of lysozyme in time dependent fashion at 90 °C with 4.4 mg/ml protein concentration^38^. This report was intriguing because higher concentration (>300 mM) of arginine have been shown to induce exclusion of proteins from solution^39^. To explore the effect of arginine and benzyl alcohol on temperature induced lysozyme association, we repeated DLS experiments with different amounts of arginine and benzyl alcohol in 20 mg/ml lysozyme solution (Fig. 4a,b). Up to 300 mM of arginine no intermediate state was observed and higher amounts of arginine led to induction of association of lysozyme (Fig. 4a). Similarly, low amounts of benzyl alcohol (1–2% v/v) clearly diminished the intermediate formation and higher concentration of benzyl alcohol induced association of protein molecules which is further enhanced with increase in temperature (Fig. 4b). Importantly, all the final stage associations, even in the presence of arginine and benzyl alcohol, showed fibril shape capable of exhibiting apple-green fringe pattern upon staining with Congo red. This confirmed that the increase in temperature eventually leads to amyloid fibril formation, and arginine or benzyl alcohol can just retard the formation of intermediate stage (*this has been discussed again below*).

Based on the DLS results, we attempted to crystallize lysozyme in buffers containing arginine and benzyl alcohol at 45 °C (Table 2). Interestingly, spherulite formation was not observed in presence of these small molecules. Very importantly, in crystals two arginine molecules were found directly bound to monomeric lysozyme, although with low occupancy and high disorder (Fig. S8A–F). One arginine was bound to residues Asn113, Lys116, Gly117 and Thr118 and second arginine was interacting with residues Gly4, Arg5 and Cys6. Interestingly, second arginine in our crystal structure was present on the same position as reported by earlier by Len *et al.*, in HEWL crystals in the presence of 0–500 mM arginine at 20 °C^40^ (Tables S3 and S8). Similarly data analysis and refinement of crystals grown in the presence of benzyl alcohol had only one chain of lysozyme with one molecule of benzyl alcohol was bound to its active site (Fig. S8G–I). It was interesting to find that the interacting residues which hold these small molecules in place were found to be common in enabling protein-protein interactions in the dimeric and trimeric form (Table S3). Additionally, superimposition of arginine and benzyl alcohol bound structures on the heat induced dimer and trimer confirmed that the bound molecules are located in interaction interface (Fig. 4e,f). Taking together the DLS and crystallography results, it appears that the phenomenon by which arginine or benzyl alcohol inhibit low order association in crystals may or may not extend into solution. Possibly, the increase in temperature reduces affinity of these ligands to lysozyme and/or increased diffusion dynamics dislodges these molecules. It is very likely a complex summation of events in solution, but it remains clear from our repetitive attempts of crystallization of lysozyme at increased temperatures in presence of these molecules that no spherulites are formed indirectly supporting that the dimeric and trimeric states of lysozyme are precursors and “on pathway” ensembles of temperature induced amyloid formation by lysozyme.

**Figure 5:**
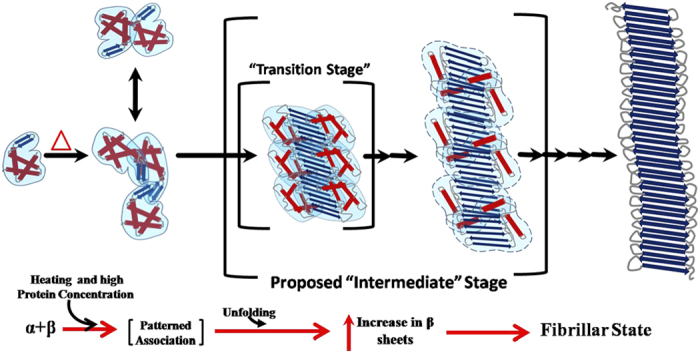
Schematic representation of the mechanism proposed by us that association of native globular proteins precede loss of native structure, which then associate to form large order species with gain in β-sheet architecture. (Blue arrows and red cylinders in our schematic represent β-sheets and α-helical order).

Another important aspect was the stability of the lysozyme protein under increased temperatures during crystallization attempts. This was particularly of value since Lara *et al.* showed hydrolysis of lysozyme at pH 2 and temperatures as high as 90 °C^6^. Denaturing and native PAGE results showed us that lysozyme in crystalline outgrowths and core of spherulites do not undergo any degradation (Fig. S9). On the other hand, heating of lysozyme in solution (20 mg/ml pH 3.8) showed that some degradation starts after 60 °C, but no detectable degradation occurs in protein at or below 60 °C up to 72 hours (Figs S10 and S11). These results confirmed that the formations of spherulites described in our study are not due to hydrolysis of the lysozyme protein. Gaining visual insight into the pro-, pre- and amyloid organization, particularly those formed by full-length proteins have remained a challenge. Incremental insights, mainly by indirect methods, have helped us conjure a picture of what probably happens when perfectly globular protein like lysozyme transforms into a building block of β-amyloid. Precedence till now, teaches us that non-native shapes of globular protein including lysozyme associate to gain cross β-sheet structures to eventually form β-amyloid fibrils^2^,^3^. It remains unclear how the partitioning of charged residues on surface and hydrophobic core gets disturbed or redistributed to achieve non-native structures, particularly when a good portion of lysozyme also forms α-helical structures. Non-specific changes in this partitioning might lead to non-native structures which might just aggregate. Physiologically, or in cellular environment, local concentration of amyloid forming proteins is actually higher than what are usually considered during representative biophysical experiments (Table S1). This could be one limiting factor in correlating observed biophysical results with biology. In our experiments, involvement of higher concentration of lysozyme led to this clear observation. Moreover, crystallization experiments also showed that lysozyme could exist in multiple organization states. Our experiments conclude that prior to loss of native structural content, low order associations occur when lysozyme is exposed to higher temperatures (Fig. 5). Probably, the associated states provide that solvent-shielded environment which allows restructuring to occur in a specific manner without leading to rapid misfolding or initiation of random aggregation. Overall, our communication provides a fresh look at the existing problem and our approach can be extended to other systems where a soluble protein/peptide transforms into amyloid organization.

## Experimental Section

### Protein Samples for Experiments

For this work, high quality hen egg white lysozyme was purchased from Biomatik Company (Wilmington, DE USA). Initial stock solutions were prepared by mixing the protein in 50 mM NaOAc buffer pH 3.8 containing 150 mM NaCl at concentrations ~30 mg/ml and dialyzed overnight against same buffer with three changes. Monomeric protein was then purified from any aggregation/associated versions using gel filtration chromatography (Sephadex200 column attached to Bio-Rad FPLC). Such purified lysozyme was concentrated to required concentrations using membrane concentrators (AMICON, Ireland). Protein concentrations were estimated by performing mass based dilution of stock samples using A_280/1mg/ml_ = 2.5 (Hitachi UV-Vis instrument U2900, USA). After each concentration (and routinely), lack of aggregation was confirmed by FPLC profile and (dynamic and multi-angle) light scattering experiments. For experiments involving variation in NaCl concentration in buffer, the changes were induced by overnight dialysis with five changes against desired buffer composition.

### Dynamic Light Scattering

All DLS experiments were carried out using DelsaNanoC instrument (Beckman Coulter, USA) equipped with built-in temperature control. All light scattering data were acquired at 165° using thermostated microcell. Diffusion coefficient values were measured for different concentrations of lysozyme as a function of protein concentration, time, temperature and salt concentration in buffer. Temperature ramping was done at 1°C/min and sample was equilibrated at the study temperature for 5 min. prior to data collection. Each experiment was done in triplicate and average diffusion coefficient values were considered for interpretation.

### Size Exclusion Chromatography-Multi Angle Light Scattering

The molecular weights (weight averages) of the lysozyme samples were determined by using inline SEC-MALS system. Acetate buffer, pH 3.8 and NaCl 450 mM was pumped at a flow rate of 0.5 ml/min through a SHODEX PROTEIN KW-800 column connected to WATERS HPLC system equipped with 515HPLC pump, 2489UV/Vis detector and 2707 autosampler. Protein concentration was kept at 20 mg/ml and 100 μl of sample was loaded into the column in each case. Each lysozyme sample was incubated for 10 minutes at the temperature range from 25 °C to 80 °C with 5 °C intervals, before injecting into the column (maintained at 25 °C). The samples were monitored using inline MALS detector DAWN 8^+^ equipped with 50 mW GaAs linearly polarized laser with a wavelength of 658 nm (Wyatt Technology, Santa Barbara, USA). The system has evenly positioned 8 detectors between 25° and 155°. Output signals from MALS detector were imported into ASTRA VI software for data processing. Apparent weight average molecular weights were obtained using the Debye plot method.

### Setting up Protein Crystallization

Purified lysozyme was concentrated to ~40 mg/ml for crystallization set-ups. It was confirmed by light scattering and FPLC that the concentration step did not lead to any aggregation in stock sample. Solutions containing 50 mM NaOAc buffer (pH 3.8) with NaCl gradient from 0.9 to 1.4 M were prepared as mother liquids during crystal setup. Crystals were grown by vapor diffusion using hanging drop method in 24 well XLR plates containing equal volume of protein (3 μl) and mother liquid (3 μl) on siliconized cover slips (Blue Star 18 mm). The plates were then transferred to vibration free RUMED unit maintained at desired temperature for crystal growth. Screening of the plates was done on alternate days to check crystal growth. Three types of crystals appear at different time spans *viz*. tetragonal crystals after 2 days, spherulites start forming after 2–3 days and elongated needle like crystals emerging out of white spherulite “cores” were spread all over the crystallization drop in a week and orthorhombic crystals formed after 10 days. Most drops incubated at 45 °C contained both tetragonal as well as needle like crystals.

### X-ray Diffraction Data Collection

Diffraction data of HEWL crystals were collected on an in-house MAR 345dtb image plate detector mounted on a RIGAKU MicroMax-007HF rotating-anode X-ray generator (λ = 1.5418) operated at 40 kV and 30 mA. Crystals were soaked in cryo-protectant solution containing 10–20% glycerol added to the corresponding mother liquid before diffraction. Intensity data from all crystals were collected at 100 K using OXFORD cryostream.sample to detector distance was 140 mm for normal crystals grown at 45 °C, while this distance for needle-like crystals was 175 and 200 mm. Each frame was recorded for 20 seconds in case of normal crystals, while exposure time was 15 minutes for needle-like crystals with 1° oscillation during recording of each image in each case. Diffraction data processing including intensity integration and scaling was done using HKL2000 suit of programs^41^. All the crystals belonged to orthorhombic space group.

### Structure Refinement

Initial structure determination for the crystals was done by molecular replacement method with program MOLREP^42^ from CCP4i suit using 1BGI as a search model. Cell parameters for the needle-shaped crystals at 45 °C were different, thus before proceeding with refinement, the number of chains in the asymmetric unit was determined by using Matthews Coefficient program^43^ of CCP4i suite of programs. The space group of these crystals was solved by PHASAR program^42^,^44^ using 1BGI as a search model. Initial models of all crystals were refined by rigid body refinement using REFMAC5^42^ followed by restrained refinement. Model building and further refinement was done by recursive use of COOT^45^ and PHENIX programs^46^ till the models were completely built. Addition of solvent molecules present in the solution began at the stage at which R_w_ value reached around 0.25 in each case. Molecules were added to electron densities where F_o_ – F_c_ map had more than 3σ value above the mean and the 2F_o_ – F_c_ map showed density at 1σ level, forming at least one hydrogen bond with protein or other solvent atom.

### Transmission Electron Microscopy (TEM)

Thin noncrystalline spikes emerging out of core of spherulites (formed in the crystallization drops at 45 °C) were abstracted. These spikes were transferred to droplets of mother liquid (placed on coverslips) for washing and 40 μl solution from these droplets was placed on 200-mesh carbon coated copper grids (purchased from EMS, Hatfiled, USA) for 20 minutes to allow the spikes settle on the grid mesh. Grids were dried and soaked in double distilled water for dissolving salt crystals if formed on the grids and then dried again. Prior to TEM studies samples on the grids were negatively stained with filtered 1% (w/v) uranyl acetate solution as described by Brenner and Horne^47^. After 20–30 seconds, excess of stain was withdrawn with Kimwipe filter paper and the grids were left for drying. Dried grids were then analyzed under Jeol Jam-2100 transmission electron.

### Fourier Transformation Infrared Spectroscopy

All experiments were carried out using Bruker Vertex 70 spectrometer equipped with a liquid nitrogen cooled Mercury Cadmium Telluride (LN-MCT) detector. Each spectrum was generated by averaging 120 scans, measured with a resolution of 2 cm^−1^. Spectra were collected at temperature range of 20–80 °C at intervals of 5 °C, using specially designed cell with CaF_2_ windows on Bio-ATR II stage and temperature was controlled by water bath (Thermo Scientific, DC10-K20 thermostat) with a precision of ±0.02 K. Transmission spectra were obtained from lysozyme solutions at varying temperatures with protein concentration of 20 mg/ml in 50 mM NaOAc buffer pH 3.8 and ~700 mM NaCl. Each sample spectra was analyzed after subtracting buffer contribution under identical conditions. Second derivative of Amide I stretching frequencies (1700–1600 cm^−1^) was performed using OPUS software to identify different contributions from different secondary structures. Deconvulation results were reconfirmed using Origin 8.0 software.

### Staining

To confirm that the higher order associations formed after thermal treatment of samples are amyloids, Congo red and Thioflavin T staining based birefringence formation experiments were carried out with the protein samples obtained after scattering experiments using the protocol described by Nilsson^48^. Briefly, the samples were spread on glass slides as a thin film and were left for air drying. Saturated amount of NaCl was dissolved in a solution of 80% ethanol: 20% distilled de-ionized (DDI) water. The solution was stirred and excess of salt was filtered out. Saturated amount of Congo red was dissolved in the resulting solution and filtered using 0.22 μm syringe filter to obtain final staining solution. Staining solution was placed on the protein film of air dried glass slides. Excess of solution was removed by adsorption. The slides were then examined under polarization microscope for the expected apple green birefringence (BX-51, Olympus). Thioflavin T staining is also done using protocol described by Nilsson^48^.

## Additional Information

**How to cite this article**: Sharma, P. *et al.* Characterization of heat induced spherulites of lysozyme reveals new insight on amyloid initiation. *Sci. Rep.* **6**, 22475; doi: 10.1038/srep22475 (2016).

## Supplementary Material

Supplementary Information

## Figures and Tables

**Table 1:** Data collection statistics from the crystals of lysozyme at 45 °C are summarized below.

	Monomer	Dimer	Trimer
Temperature (Crystallization)	45 °C	45 °C	45 °C
Data Collection			
Wavelength (Å)	1.5418	1.5418	1.5418
Resolution (Å)	50–1.71	50–1.95	50–2.65
Space group	*P*2_1_2_1_2_1_	*P*2_1_2_1_2	*P*2_1_2_1_2_1_
Unique Reflections	13859	16785	10188
Unit cell parameters			
a (Å)	30.01	31.62	31.47
b (Å)	55.95	66.44	92.35
c (Å)	72.48	104.45	114.24
α = β = γ	90°	90°	90°
Completeness (%)	94.5(99)	89.6(97)	93(83)
R merge (%)	3.4(6.1)	6.2(29.4)	18.8(51.5)
Average *I/σ(I)*	32.35(20.9)	20.58(3.98)	6.5(2.00)
Refinement			
R_work_(%)	18.22	18.13	20.60
R_free_(%)	22.98	22.52	30.45
Solvent content	42.07	35.73	36.29
No. of chains	A	A, B	A, B, C
r.m.s.d. from ideality			
Bonds (Å)	0.006	0.007	0.008
Angles (°)	0.967	1.104	1.257
Wilson B-factor (Å^2^)	18.24	21.52	28.9
Ramachandran (%)			
Favoured	89.4	88.5	82.3
Allowed	10.6	11.5	17.7
**PDB accession code**	**4D9Z**	**4R0F**	**4DC4**

Values in parentheses refer to the highest resolution shell.

**Table 2:** Data collection statistics from the crystals of lysozyme with arginine and benzyl alcohol in buffer and formed at 45 °C are summarized below.

	Lysozyme with Arginine	Lysozyme with Benzyl alcohol
Temperature(Crystallization)	45 °C	45 °C
Data Collection		
Wavelength (Å)	1.5418	1.5418
Resolution (Å)	50–1.83	50–1.75
Space group	*P*2_1_2_1_2_1_	*P*4_3_2_1_2
Unique Reflections	11363	11975
Unit cell parameters		
a (Å)	30.52	78.17
b (Å)	55.51	78.17
c (Å)	72.43	37.14
α = β = γ	90°	90°
Completeness (%)	99.7(99.8)	98.76(99)
R merge (%)	4.1(11)	3.8(12.3)
Average *I/σ(I)*	36.6(17.4)	26.58(5.24)
Refinement		
R_work_(%)	18.7	18.78
R_free_(%)	24.6	21.02
Solvent content	42.54	43.21
No. of chains	A	A
r.m.s.d. from ideality		
Bonds (Å)	0.007	0.008
Angles (°)	1.042	1.113
Wilson B-factor (Å^2^)	24.26	27.26
**PDB accession code**	**4EOF**	**4II8**

Values in parentheses refer to the highest resolution shell.
