# Supplementary material for: Characterization of heat induced spherulites of lysozyme reveals new insight on amyloid initiation
Source: Sci Rep. 2016 Mar 1;6:22475. doi: 10.1038/srep22475 (PMC4772699; doi:10.1038/srep22475)
Supplement: Supplementary Information [file srep22475-s1.pdf]

Supplementary Data

Characterization of heat induced spherulites of lysozyme reveals new insight on amyloid initiation

Pankaj Sharma, Neha Verma, Pradip Kumar Singh, Suresh Korpole and Ashish*
CSIR-INSTITUTE OF MICROBIAL TECHNOLOGY, CHANDIGARH, INDIA

**Address correspondence to:* Ashish, CSIR-Institute of Microbial Technology, Sector 39-A, Chandigarh INDIA 160036 Phone: +911726665472; Fax: +911722690685; Email: ashgang@imtech.res.in

Table S1 Summary of protein concentrations used for studying amyloid formation by hen egg white lysozyme (and human lysozyme) using different biophysical methods.

S.No	Paper	Year	Protein*	Protein conc.
1	Instability, unfolding and aggregation of human lysozyme variants underlying amyloid fibrillogenesis (Booth, Sunde *et al.* 1997).	1997	HL	[1]Cry.-10mg/ml<br>[2]IR- 5mg/ml<br>[3]CD-0.22mg/ml<br>[4]F- 0.2mg/ml<br>[5]MS-0.28μg/ml
2	Local cooperativity in the unfolding of an amyloidogenic variant of human lysozyme (Dhulesia, Cremades *et al.* 2010).	2002	HL	8.69 mg/ml
3	Reduced Global Cooperativity is a Common Feature Underlying the Amyloidogenicity of Pathogenic Lysozyme Mutations (Dumoulin, Canet *et al.* 2005).	2005	HL	0.1 mg/ml<br>[3]CD-0.2 mg/ml<br>[4]F-0.035mg/ml
4	Identification of the core structure of lysozyme amyloid fibrils by proteolysis (Frare, Mossuto *et al.* 2006).	2006	HL	14mg/ml,<br>10mg/ml
5	Amyloid Protofibrils of Lysozyme Nucleate and Grow Via Oligomer Fusion (Hill, Robinson *et al.* 2009).	2009	HEWL	17mg/ml
6	Structure of amyloid fibrils of hen egg white lysozyme studied by microbeam X-ray diffraction (Yagi, Ohta *et al.* 2009).	2009	HEWL	0.01mg/ml
7	Characterization of Oligomeric Species on the Aggregation Pathway of Human Lysozyme (Frare, Mossuto *et al.* 2009).	2009	HL	[4]F-0.0336mg/ml<br>[3]CD-0.098mg/ml<br>[6]TEM-9mg/ml
8	Local Cooperativity in an Amyloidogenic State of Human Lysozyme Observed at Atomic Resolution (Dhulesia, Cremades *et al.* 2010).	2010	HL	[3]CD-0.28mg/ml,<br>[7]DSC-0.56mg/ml
9	Population of Nonnative States of Lysozyme Variants Drives Amyloid Fibril Formation (Buell, Dhulesia *et al.* 2011).	2011	HL	0.98mg/ml

10	Unfolding and aggregation of lysozyme: A thermodynamic and kinetic study by FTIR spectroscopy (Sassi, Giugliarelli *et al*. 2011).	2011	HEWL	30mg/ml, 120mg/ml
11	Spatial Extent of Charge Repulsion Regulates Assembly Pathways for Lysozyme Amyloid Fibrils (Hill, Miti *et al.* 2011).	2011	HEWL	0.42mg/ml

* HL - Human lysozyme

HEWL- Hen egg white lysozyme

1. Cry. - Crystallization
2. IR - Infrared Spectroscopy
3. CD - Circular Dichroism
4. F - Fluorescence Spectroscopy
5. MS - Mass spectroscopy
6. TEM - *Transmission electron microscopy*
7. DSC - *Differential scanning calorimetry*

Table S2

Table of different states of lysozyme at different temperature and salt conditions are tabulated below.

NaCl / Temperature	0.9-1.2M	1.2-1.3M	1.3-1.4M	Above 1.4M
**35°C**	Tetragonal crystals	Tetragonal crystals	Tetragonal crystals	Aggregates
**40°C**	Tetragonal crystals	Tetragonal/ Orthorhombic crystals + Small Spherulites	Spherulites	Aggregates
**45°C**	Tetragonal crystals	Tetragonal/ Orthorhombic crystals + Spherulites	Spherulites + Aggregates	Aggregates
**50°C**	Spherulites + Aggregates	Aggregates	Aggregates	Aggregates
**55°C**	Aggregates	Aggregates	Aggregates	Aggregates

Table S3

Table of residues involved in interactions between weakly associated lysozyme dimer and trimer. Among these the highlighted residues (bold and underlined) are also involved in interaction with arginine.

**Lysozyme Dimer 3R0F**		**Lysozyme Trimer 4CD4**			
Hydrogen bonding	Non-bonded contacts	Hydrogen bonding		Non-bonded contacts	
Chain A–Chain B	Chain A-Chain B	Chain A-Chain B	Chain B-Chain C	Chain A-Chain B	Chain B-Chain C
Arg14-Asn37	Arg14 - Val2	Asn77-Arg112	Arg73-Gly49	His15-**Thr118**	Trp62-Pro70
Asp87-**Arg5**	Arg14 - Asn37	Ala82-Arg114	Arg73-Arg68	Cys76-Arg112	Trp62-Arg68
	His15 - Val2	Asn93-Arg112	Gly102-Arg73	Asn77-Arg112	Trp62-Gly67
	Pro79 - Arg128	Asn93-**Lys116**		Ile78-**Asn113**	Trp63-Pro70
	Ser81 - Arg128			Ala82-Arg114	Arg73-Arg68
	Asp87- Gly4			Ala82-**Asn113**	Arg73-Gly49
	Asp87- **Arg5**			Ser85-Arg114	Lys97-Asp48
	Thr89 - **Gly4**			Asp87-Arg114	Asp101-Arg61
	Thr89 - Val2			Thr89-**Asn113**	Asp101-Asp48
				Thr89-Arg114	Gly102-Trp62
				Thr89-**Thr118**	Gly102-Arg73
				Thr89-**Lys116**	Asn103-Gly71
				Ala90-**Asn113**	
				Ala90-Arg114	
				Asn93-**Lys116**	
				Asn93-Arg112	

Table S4

Table of atoms of residues involved in hydrogen bonding between the dimeric and trimeric crystalline state of lysozyme are tabulated below.

Lysozyme Dimer PDB ID: 3R0F									
Atom 1					Atom 2				
S.No.	Atom Name	Residue Name	Residue Number	Chain	Atom name	Residue Name	Residue Number	Chain	Distance (Å)
1.	NH2	ARG	14	A	O	ASN	37	B	3.13
2.	OD2	ASP	87	A	N	ARG	5	B	3.22
**Lysozyme Trimer PDB ID: 4CD4**									
1.	O	ASN	77	A	NH1	ARG	112	B	3.19
2.	O	ALA	82	A	NH2	ARG	114	B	2.78
3.	ND2	ASN	93	A	O	ARG	112	B	3.29
4.	ND2	ASN	93	A	O	LYS	116	B	2.57
5.	NH1	ARG	73	B	O	GLY	49	C	3.27
6.	NH1	ARG	73	B	O	ARG	68	C	2.96
7.	O	GLY	102	B	NH1	ARG	73	C	3.29

Table S5

Comparison of previously resolved dimeric associations of lysozyme with our heat-induced dimeric and trimeric association (*cited references are below*).

PDB Code	Space Group	Interchain H- Bonding	RMSD	Crystallization conditions	Summary
1B2K	$P2_1$ Monoclinic	119-87	4.04	Lysozyme 50mg/ml, 0.14M NaI, pH 4.5, 291K.	Monoclinic crystals of lysozyme contain two protein molecules in an asymmetric unit. These molecules are independent and oriented parallel to each other. Ions I^-, NO_3^- and SCN^- are present between the two lysozyme molecules. These ions interact with both lysozyme molecules (19).
1HF4	$P2_1$ Monoclinic	119-87	18.72	Lysozyme 10mg/ml, 0-290mM $NaNO_3$, pH 5, 293K.	
1LCN	$P2_1$ Monoclinic	119-87	18.82	Protein7.7 mg/ml, 50mM KOAc, pH 4.5, 190mM KSCN, 291K.	
2D4K	$P2_1$ Monoclinic	119-87<br>116-77	18.85	2% lys., 10% NaCl, 5% 1-propanol, pH 7.6, 313K	The mechanism of phase transition of protein crystals was elucidated by studying the structural changes in relation to change in solvation. Asymmetric unit contains two independent protein molecules (20).
2D4I	$P2_1$ Monoclinic	119-87<br>116-77	18.78	1% protein, 2% $NaNO_3$, pD 4.5, 298K.	
1LJ3	$P2_1$ Monoclinic	119-87<br>116-77	18.73	0.1M NaOAc pH 4.6, 298K, 0.44M $NaNO_3$	Monoclinic lysozyme crystals were grown as they contain two crystallographically independent molecules with slightly different packing environments. This helps in accessing the effect of normal and stabilizing additives (sucrose, trehalose, sorbitol and glycerol) on structure and hydration of lysozyme molecules (21).
1LJ4	$P2_1$ Monoclinic	119-87<br>116-77	18.74	0.1M NaOAc pH 4.6, 293K, 0.44M $NaNO_3$	
1LJE	$P2_1$ Monoclinic	119-87	18.77	0.1M NaOAc pH 4.6, 298K, 0.44M $NaNO_3$, 0.29M sucrose.	
1LJF	$P2_1$ Monoclinic	119-87<br>116-77	18.77	0.1M NaOAc pH 4.6, 293K, 0.44M $NaNO_{3,}$ 0.29M sucrose.	
1LJG	$P2_1$ Monoclinic	119-87<br>116-77	18.74	0.1M NaOAc pH 4.6, 298K, 0.44M $NaNO_{3,}$ 0.54M glycerol.	
1LJH	$P2_1$ Monoclinic	119-87<br>116-77	18.76	0.1M NaOAc pH 4.6, 298K, 0.44M $NaNO_{3,}$ 0.54M glycerol.	
1LJI	$P2_1$ Monoclinic	119-87<br>116-77	18.73	0.1M NaOAc pH 4.6, 298K, 0.44M $NaNO_{3,}$ 10% sorbitol.	
1LJJ	$P2_1$ Monoclinic	119-87	18.73	0.1M NaOAc pH 4.6, 298K, 0.44M $NaNO_{3,}$ 0.29M trehalose.	
1LJK	$P2_1$ Monoclinic	119-87<br>116-77	18.79	0.1M NaOAc pH 4.6, 298K, 0.44M $NaNO_{3,}$ 0.44M trehalose.	
1LKR	$P2_1$ Monoclinic	119-87	18.81	1% protein, 200 mM acetate buffer PH 8, 5%NaI.	Two lysozyme molecules are present in an asymmetric unit. I^- ions are present between protein molecules, it links both molecules thus stabilizes the monoclinic form. Lysozyme molecules are oriented parallel to

					each other (22).
1LYS	$P2_1$ Monoclinic	113-81	18.82	10% NaCl, pH 7.6, 5% 1-propanol, 313K	The two molecules are parallel to each other (23).
1UCO	$P2_1$ Monoclinic	119-87<br>116-77	18.74	Data not available	Lysozyme crystals were transformed from native to low humidity forms to investigate the effect of humidity on hydration, mobility and enzyme action of lysozyme (24).
1V7T	$P2_1$ Monoclinic	37-73	14.49	1%protein, 2% $NaNO_3$, 10mM NaOAc, 313K, pD 4.5.	A triclinic crystal was transformed into a new triclinic crystal. Transformed crystal contains two independent molecules of lysozyme oriented parallel to each other (25).
1VDP	$P2_1$ Monoclinic	119-87<br>116-77<br>112-74	18.78	Data not available	Data not available
3LYT	$P2_1$ Monoclinic	-----	18.70	Data not available	Data not available
4LYT	$P2_1$ Monoclinic	119-87	18.75	Data not available	Data not available
5LYM	$P2_1$ Monoclinic	119-87	18.79	Data not available	Data not available
1RCM	$P2_12_12_1$ Orthorhimbic	66-39<br>81-41<br>65-41<br>41-81<br>41-65<br>39-66<br>43-68<br>68-43	14.73	Protein 9mg/ml, 7% NaCl, 50mM NaOAc.	Reduction of disulfide bond between cyt. 6 and cyt. 127 displace the C-terminal segment from its conformation in the native protein, this make lysozyme a ubiquitination target. Asymmetric unit contains two lysozyme molecules oriented antiparallel to each other (26).
3VFX Dimer	$P22_12_1$ Orthorhimbic	87-5<br>14-37		50mM sodium acetate, 1-1.5M NaCl, pH 3.8, temperature 318K	**Our Study**
4DC4 Trimer	$P2_12_12_1$ Orthorhimbic	A-B<br>B-C<br>77-112<br>73-49<br>93-112<br>73-68<br>93-116<br>102-73<br>82-114		50mM sodium acetate, 1-1.5M NaCl, pH 3.8, temperature 318K	**Our Study**

Table S6

Table of residues involved in interaction with arginine and benzyl alcohol bound to lysozyme and resolved in PDB IDs 4EOF (w arginine) and 4II8 (w benzyl alcohol).

**Ligand**	**Residues involved in interaction with ligand (PDB ID: 4EOF)**	**Residues involved in hydrogen bonding with ligand (PDB ID: 4II8)**
Arginine 1	Asn113,Lys116,Gly117 and Thr118	Asn113 and Gly117
Arginine 2	Gly4,Arg5 and Cys6	Cys6
Benzyl alcohol	Glu57,Asn59,Ala105 andGlu35	Glu57

Figure S1

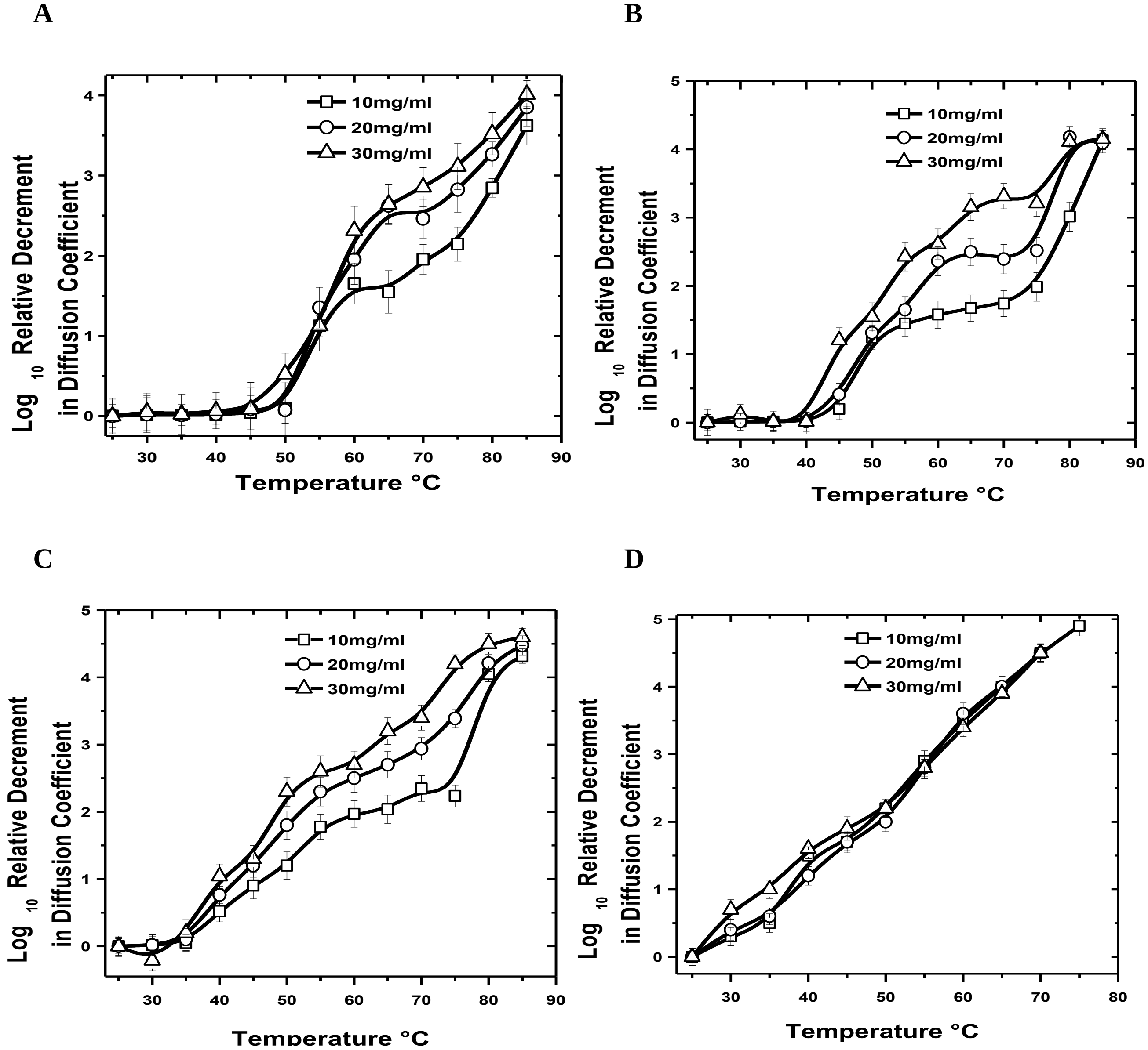

Tracking increase in particle size of lysozyme as a function of temperature, protein and salt concentration in solution. **(A)** Relative change in diffusion coefficient at 10 mg/ml (-□-), 20 mg/ml (-○-) and 30 mg/ml (-Δ-) protein concentrations on heating lysozyme from 20 to 85°C in 150 mM, **(B)** 300 mM, **(C)** 450 mM and **(D)** 600 mM NaCl at pH 3.8 is presented here. (*Lines are spline curves plotted to guide the eye on the trend*).

Figure S2

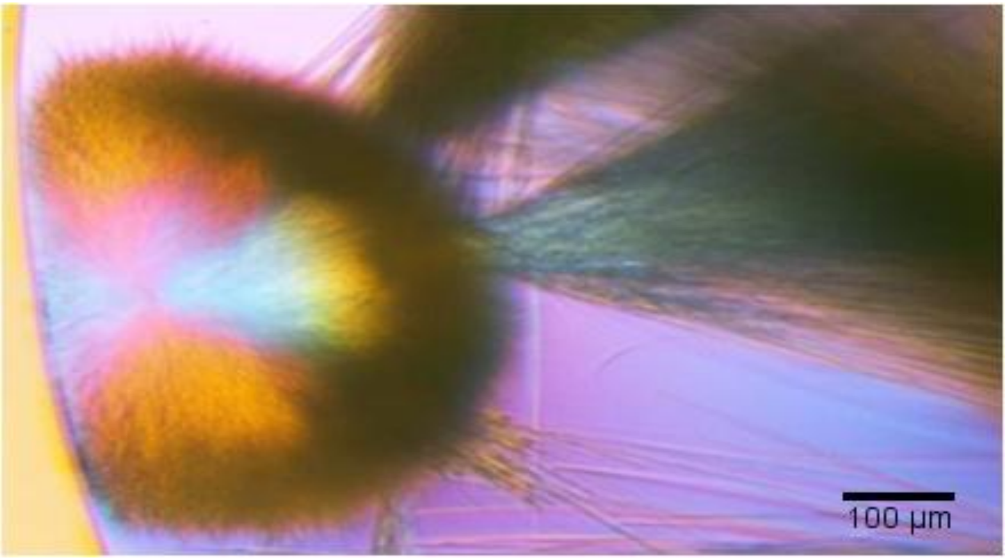

Image of HEWL spherulite: This image displays a clear Maltase-cross extinction pattern when observed between crossed polarizers. This observation shows that core of spherulites is composed of outwardly oriented fibrils.

Figure S3

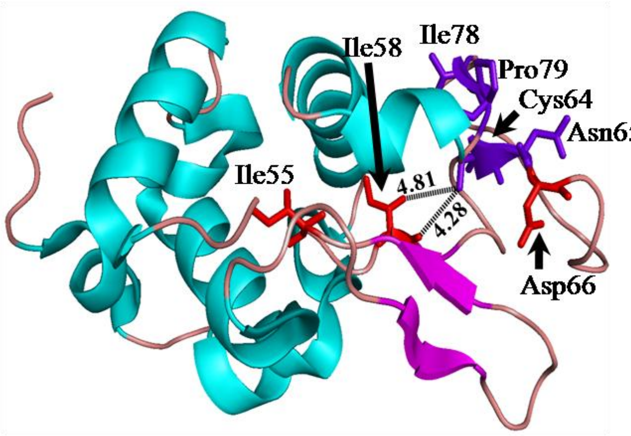

One chain of trimeric lysozyme (PDB ID 4DC4) showing residues homologous to human lysozyme responsible for amyloid formation (red) and residues involved in formation of additional β-strands in our structure (purple).

Figure S4

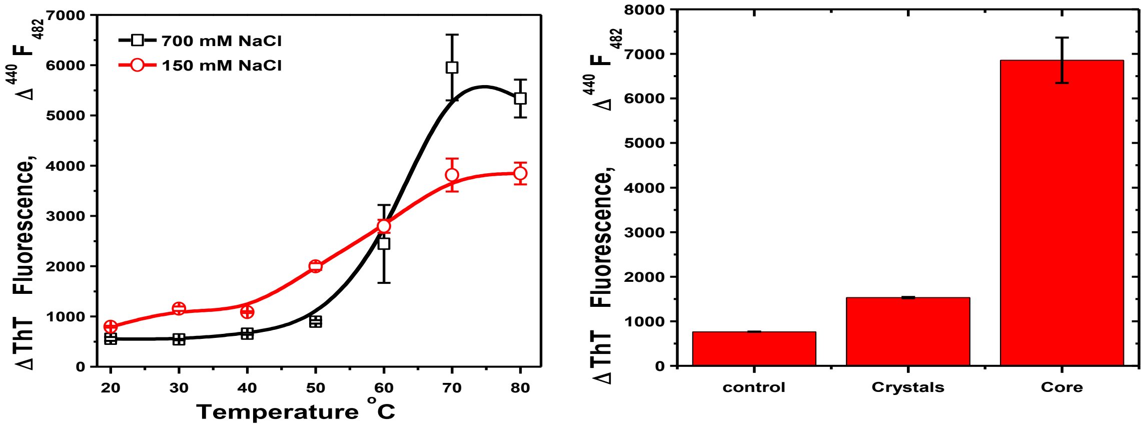

ThT staining of lysozyme samples: Lysozyme samples (20 mg/ml) were incubated for 3 days at different temperatures (20 to 80°C).

(A) A portion of the samples were stained with ThT dye, known to bind and thus stain amyloid specific structural features. Significant increase in the fluorescence with increment in temperature in the presence of 150 and 700 mM NaCl in buffer confirmed that amyloid fibrils were formed above 50°C.

(B) Also, crystalline outgrowths and core of spherulites were stained with ThT. Significant difference in the fluorescence value of the core compared to crystalline outgrowths reconfirmed that core is composed of amyloid fibrils.

Figure S5

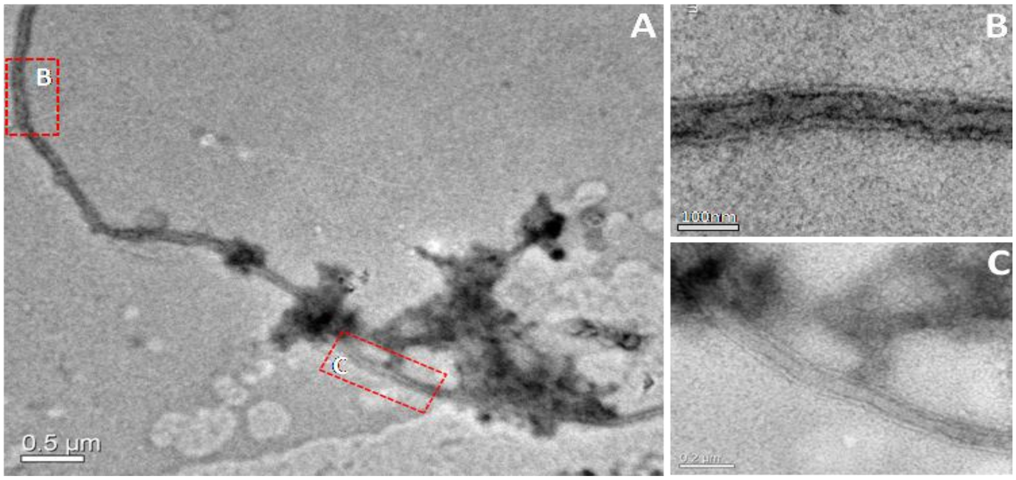

(A) TEM images of thin spikes emerging out of spherulites.
(B and C) Images show detailed architecture of fibrils on 100 and 200 nm scale, respectively.

Figure S6

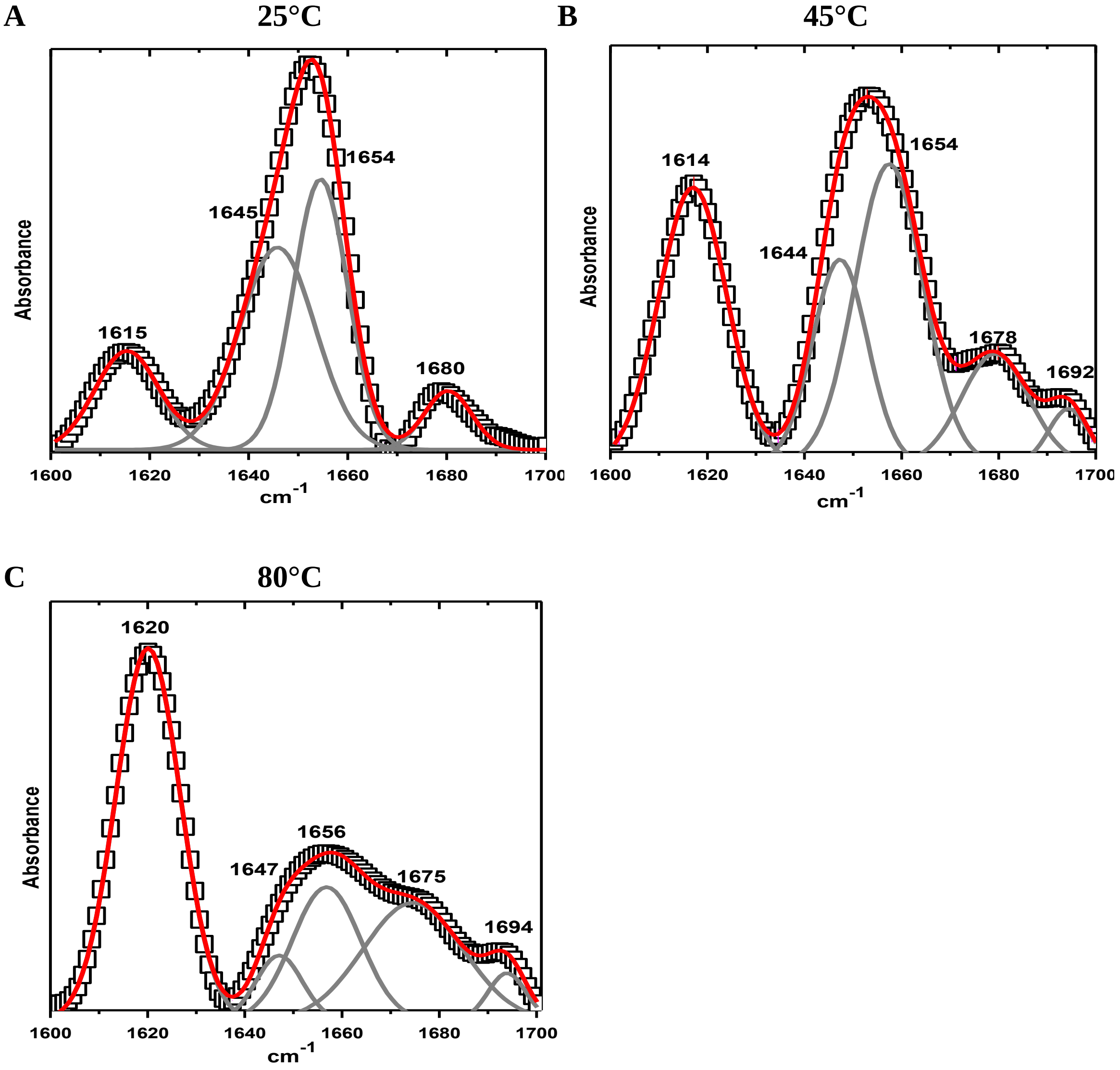

FTIR spectra for lysozyme (20mg/ml, 700 mM NaCl and pH 3.8) collected at **(A)** 25, **(B)** 45 and **(C)** 80°C. Experimental data (□), post buffer subtraction and baseline correction is shown. Curve fitting was done considering the experimental profile to be composed of multiple Gaussian peaks close to 1620, 1645, 1655, 1675 and 1690 cm^{-1}. The search and fitting process yielded peaks closer to these numbers (shown in grey lines). The addition of the curves in grey lines is shown as red line, and in all cases the summation represented the experimental data (as can be seen by the overlap). Area under the computed peaks (grey lines) for β-sheet(s) and α-helical content were used to estimate secondary structural content in the HEWL sample at the temperature studied.

Figure S7

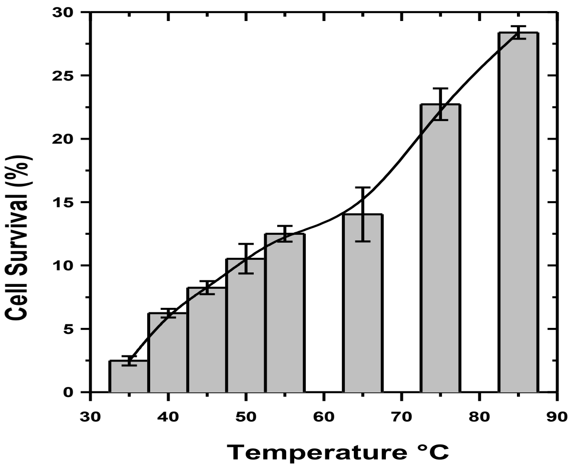

Lysozyme Activity by Lytic Assay: Purified lysozyme (~20 mg/ml) was dialyzed against filter sterilized buffer containing 500 mM NaCl and 50 mM NaOAc at pH 3.8. The dissolved lysozyme was incubated for one hour at different temperatures by gradually increasing the temperature from 35, 45, 50, 55, 65, 75 to 85°C. Protein samples were collected at each temperature for microbe lysis ability assay. *Microccocus luteus* cells were grown at 30°C in 10 ml nutrient broth and the culture was harvested at mid log phase (A_{600}≈0.6). The cells were resuspended in 1 ml of lysozyme samples, collected at above mentioned temperatures. The resultant suspension was further incubated for 30 minutes at 35°C and the lysozyme activity was measured by colony forming unit (CFU) count after overnight incubation of plates at 30°C.

The above plot shows the cell survivability in % after treating with HEWL sample earlier heated at increased temperature.

Figure S8

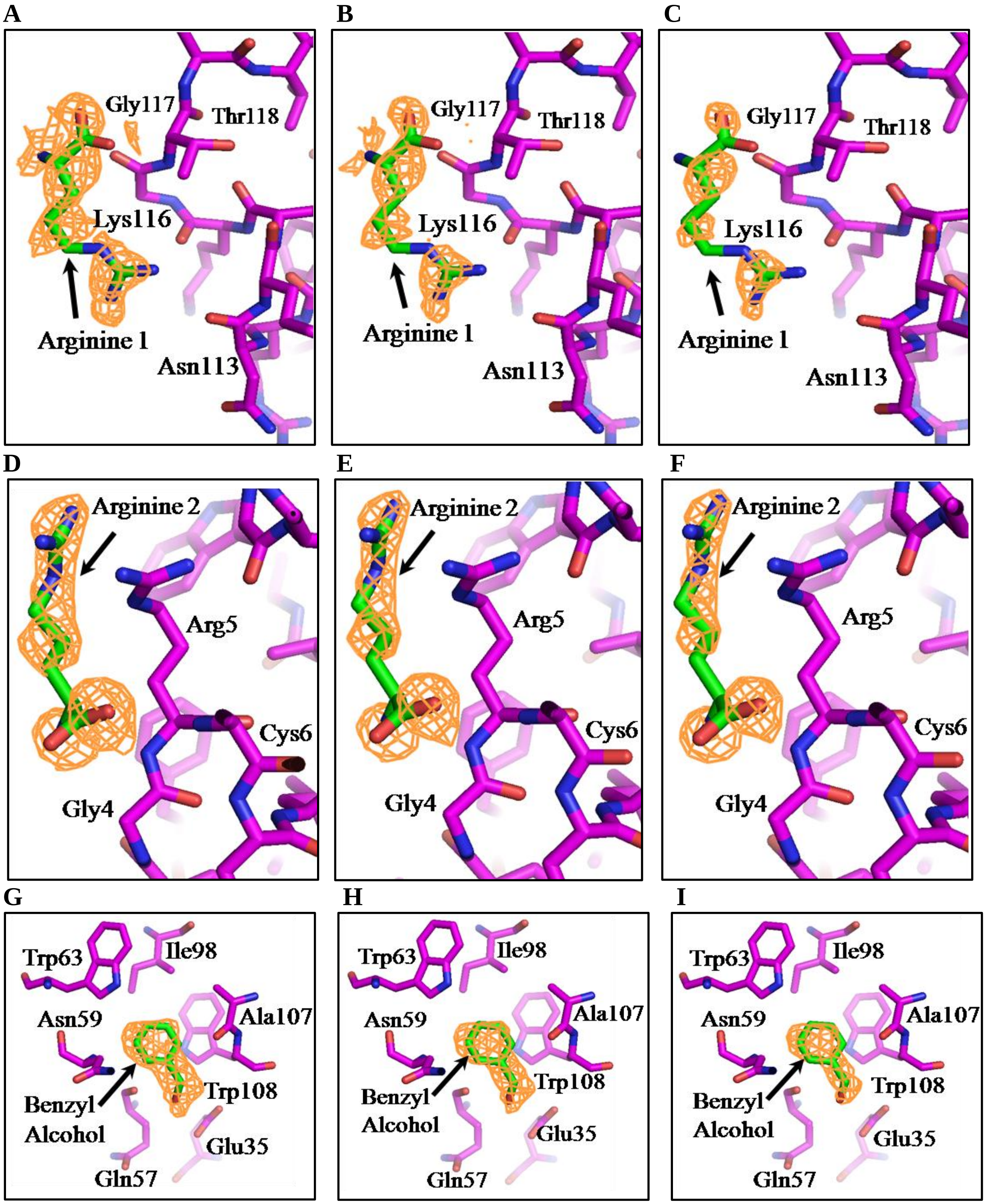

(**A**,**B**,**C**,**D**,**E and F**) Electron density maps (orange mesh) for arginine and (**G**,**H** and **I**) benzyl alcohol with lysozyme residues involved in interaction. The *F*o-*F*c maps contoured at 2.0 σ (first pannel), 2.5 σ (second pannel) and 3 σ (third pannel) are shown as orange mesh.

Figure S9

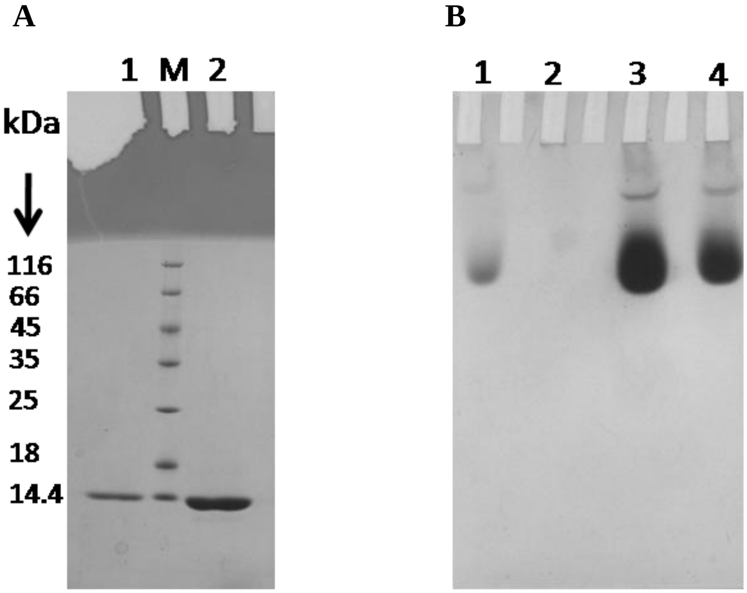

SDS-PAGE of crystalline needle shaped outgrowths and core of spherulites:

(A) SDS-PAGE of (**lane 1**) crystalline outgrowths and **(lane 2)** core of the spherulites formed during crystallization attempts of HEWL at 45°C were loaded on SDS-PAGE.

(B) Native- PAGE. Samples were: (lane 1) freshly prepared HEWL solution, (lane 2) core of the spherulites, (lane 3) tetragonal crystals and (lane 4) crystalline outgrowths from spherulites grown at 45°C.

Nothing could be seen in lane 2, probably because the association was too large to enter the gel.

These results confirmed that there was no degradation of HEWL protein during crystallization set-ups which led to spherulites described in this work.

Figure S10

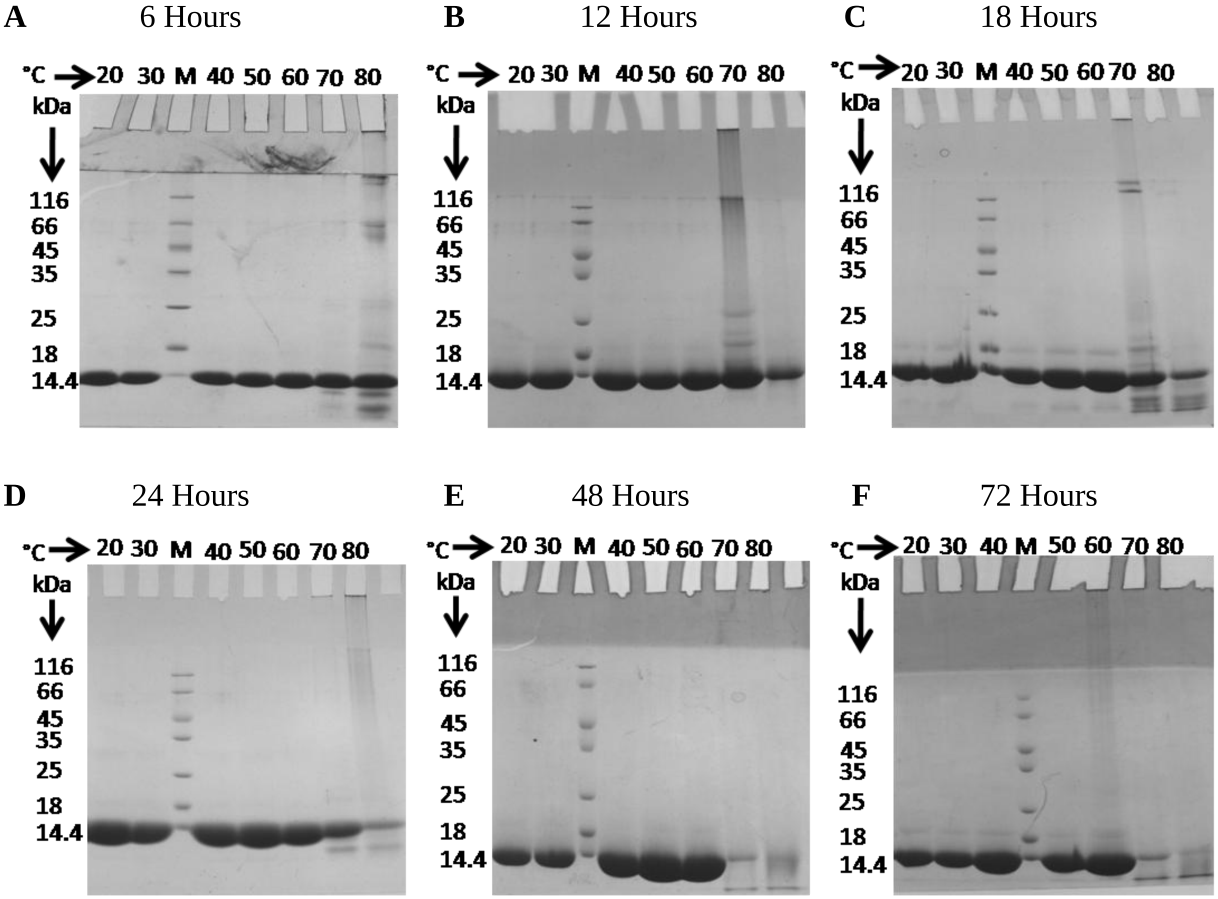

Effect of temperature on HEWL as a function of time characterized by 15% SDS-PAGE is shown here.

HEWL at 20 mg/ml in 700 mM NaCl was incubated 20 to 80°C, then 3µl of sample is loaded in each well. SDS-PAGE gels were run with samples heated for (A) 6, (B) 12, (C) 18, (D) 24, (E) 48 and (F) 72 hours.

<u>Remarkable primary observation is that no HEWL protein degradation due to heating was observed below 60°C up to 72 hours.</u>

Figure S11

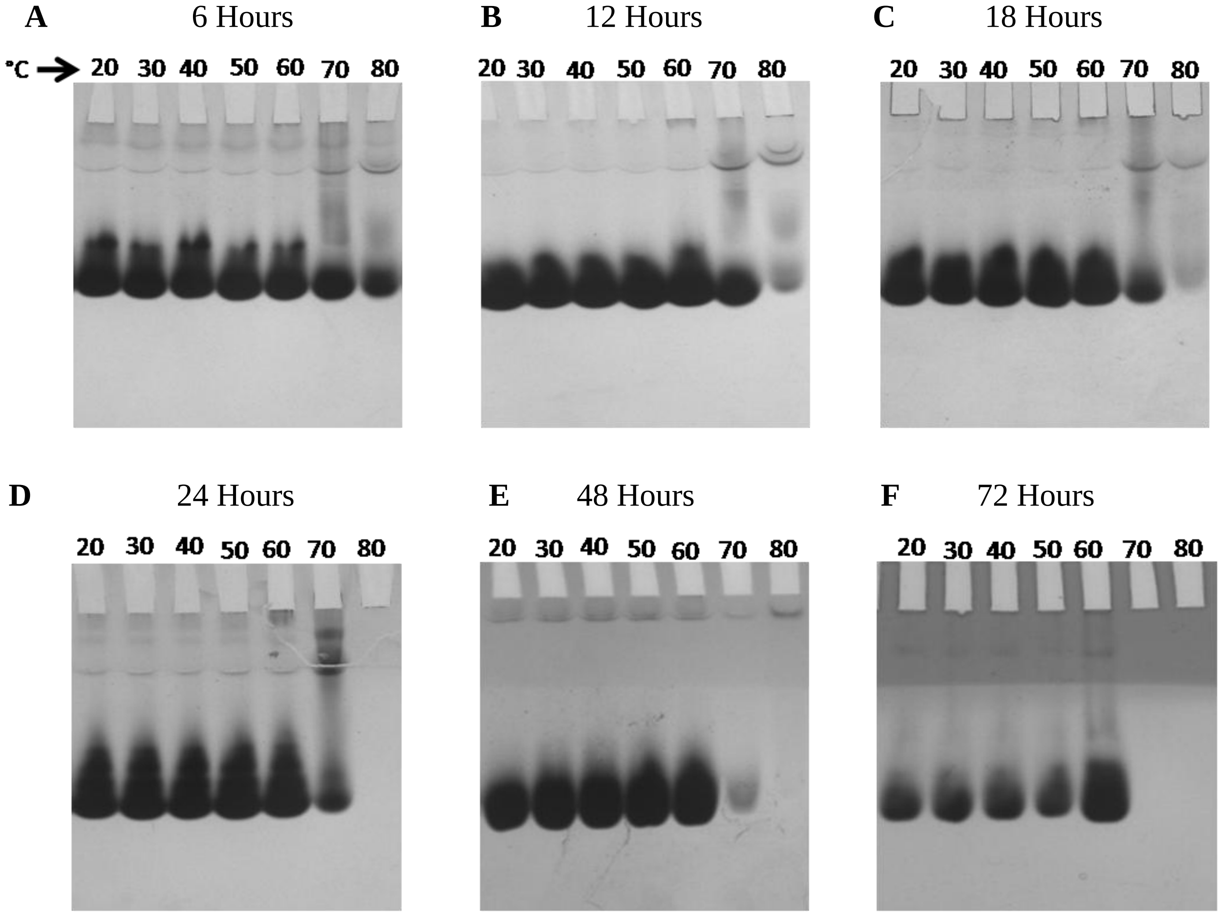

Effect of temperature on HEWL as a function of time as characterized by 10% Native-PAGE is shown here.

The 10% native-PAGE was run using the protocol similar to that of SDS-PAGE (**shown in Figure S9**) except for the absence of SDS in the loading buffer and the gel.

HEWL at 20mg/ml in 700 mM NaCl was incubated 20°C to 80°C, 3µl of sample is loaded in each well. PAGE gels were run with samples heated for (A) 6, (B) 12, (C) 18, (D) 24, (E) 48 and (F) 72 hours.

Primary observation was the observation of higher order oligomers started after 6 hours above 60°C as depicted by reduction in band intensity. After 12 hours, bands corresponding to 70°C and 80°C start disappearing as more protein units were converted to higher order oligomeric state.

Importantly, under 60°C, most of the HEWL protein was present in native monomeric state up to 72 hours of incubation. (*The tailings of the bands do suggest some low order association, but we are refraining from commenting on those as the gels are deliberately overloaded since their M/Z values would be identical*).
